# Partial Autoinformation to Characterize Symbolic Sequences

**DOI:** 10.3389/fphys.2018.01382

**Published:** 2018-10-11

**Authors:** Frederic von Wegner

**Affiliations:** ^1^Epilepsy Center Rhein-Main, Goethe University Frankfurt, Frankfurt am Main, Germany; ^2^Department of Neurology and Brain Imaging Center, Goethe University Frankfurt, Frankfurt am Main, Germany

**Keywords:** EEG microstates, information theory, entropy, mutual information, Markovianity, stationarity

## Abstract

An information-theoretic approach to numerically determine the Markov order of discrete stochastic processes defined over a finite state space is introduced. To measure statistical dependencies between different time points of symbolic time series, two information-theoretic measures are proposed. The first measure is time-lagged mutual information between the random variables *X*_*n*_ and *X*_*n*+*k*_, representing the values of the process at time points *n* and *n* + *k*, respectively. The measure will be termed autoinformation, in analogy to the autocorrelation function for metric time series, but using Shannon entropy rather than linear correlation. This measure is complemented by the conditional mutual information between *X*_*n*_ and *X*_*n*+*k*_, removing the influence of the intermediate values *X*_*n*+*k*−1_, …, *X*_*n*+1_. The second measure is termed partial autoinformation, in analogy to the partial autocorrelation function (PACF) in metric time series analysis. Mathematical relations with known quantities such as the entropy rate and active information storage are established. Both measures are applied to a number of examples, ranging from theoretical Markov and non-Markov processes with known stochastic properties, to models from statistical physics, and finally, to a discrete transform of an EEG data set. The combination of autoinformation and partial autoinformation yields important insights into the temporal structure of the data in all test cases. For first- and higher-order Markov processes, partial autoinformation correctly identifies the order parameter, but also suggests extended, non-Markovian effects in the examples that lack the Markov property. For three hidden Markov models (HMMs), the underlying Markov order is found. The combination of both quantities may be used as an early step in the analysis of experimental, non-metric time series and can be employed to discover higher-order Markov dependencies, non-Markovianity and periodicities in symbolic time series.

## 1. Introduction and background

Information theory occupies a central role in time series analysis. The concept of entropy provides numerous important connections to statistical physics and thermodynamics, often useful in the interpretation of the results (Kullback, 1959; Cover and Thomas, 2006). Despite the large number of available measures, there is no generally accepted systematic procedure for the analysis of symbolic time series, although collections of theory and methods are readily available (Daw et al., 2003; Mézard and Montanari, 2009). In metric time series analysis however, a hierarchical set of analyses and tests has been established by Box and Jenkins (Box and Jenkins, 1976). The seminal work by these authors deals with autoregressive and moving average processes, some of the most prominent Markov processes across many fields of science (Häggström, 2002). The result is a standardized procedure for analyzing continuous valued, discrete time stochastic processes (Box and Jenkins, 1976). The procedure addresses the impressive complexity of possible stochastic processes by combining semi-quantitative, visual analysis steps with a number of rigorous statistical test procedures. The first step in Box-Jenkins analysis is the visual and statistical assessment of the autocorrelation function (ACF) and the partial autocorrelation function (PACF) of the data. In particular, the order of purely autoregressive processes can be directly deduced from the PACF coefficients. For a p-th order autoregressive process, it can be shown that PACF coefficients for time lags larger than p are equal to zero, within statistical limits.

Information-theoretical time series analysis is closely linked to the theory of Markov processes, as Markov processes are defined via their temporal dependencies. The elemental case is a first-order Markov process (_*X*_*n*_)*n*∈ℤ_, for which the transition *X*_*n*_ → *X*_*n*+1_ depends on the current state *X*_*n*_ only. Due to this property, first-order Markov processes are termed memory-less, as their past does not influence transitions to future states. Markov processes of order *M* generalize this property and have transition probabilities defined by *M* past states (*X*_*n*_, …, *X*_*n*−*M*+1_), thus representing finite memory effects of the time series. Using information-theoretical methods, memory effects and temporal dependencies can be quantified. A more formal treatment of Markov processes follows in the Materials and Methods section, after introducing the necessary notation.

To assess the Markov property of a time series, and to identify the Markov order of an empirical symbol sequence, classical statistics derives a number of tests as detailed in the Materials and Methods section (Hoel, 1954; Anderson and Goodman, 1957; Goodman, 1958; Billingsley, 1961; Kullback et al., 1962). We recently used the tests developed in (Kullback et al., 1962) to characterize electroencephalographic (EEG) data transformed into a symbolic time series termed microstate sequences (von Wegner and Laufs, 2018). Using time-lagged mutual information, we could show that the symbolic four-state sequences still retain periodic features of the underlying continuous EEG signal (von Wegner et al., 2017, 2018). The aim of the present article is to introduce partial autoinformation as a measure that is complementary to the time-lagged mutual information function, in the same sense that the PACF complements the autocorrelation function in classical time series analysis of continuous random variables. In the past, we have used the term autoinformation function (AIF) for time-lagged mutual information, in analogy with the autocorrelation function (ACF) of classical time series analysis (von Wegner et al., 2017). To continue the analogy, the newly introduced measure will be termed partial autoinformation function (PAIF), because it answers the same question about the information content of a symbolic time series as the partial autocorrelation function (PACF) does about correlations. The new measure is derived based on the analogy with the PACF and theoretical connections with well-known functionals such as the entropy rate and active information storage are established. Next, we apply the AIF/PAIF approach to a number of symbolic time series ranging from Markov and non-Markov model data with known properties to simulated data representing physical systems (Ising model, abstract ion channel model) and experimental EEG microstate data. Finally, limitations and possible applications are discussed for larger state spaces and finite samples.

## 2. Material and methods

### 2.1. Autoregressive processes

To illustrate the motivation for this study, an exemplary autoregressive process is used to explain the principles of time series analysis with the (partial) autocorrelation approach. Autoregressive (AR) processes model time series of continuous random variables in discrete time (Box and Jenkins, 1976). The p-th order or AR(p) process models the dependency of *X*_*n*_ on its past via a linear combination of the p values preceding *X*_*n*_:

(1) $$\begin{array}{r} X_{n}=\phi_{1}X_{n-1}+\ldots +\phi_{p}X_{n-p}+\varepsilon_{n} \end{array}$$

where ϕ_1_, …, ϕ_*p*_ are called the autoregression coefficients and ε_*n*_ represents identically and independently distributed (iid) Gaussian noise.

The linear dependencies created by Equation (1) can be quantified by the time autocorrelation function (ACF). The ACF coefficients ρ_*k*_ of a stationary stochastic process *X*_*n*_ are defined as:

(2) $$\begin{array}{r} \rho_{k}=C\left(X_{n+k},X_{n}\right) \end{array}$$

where $C\left(X,Y\right)=\underset{i}{\sum }\frac{\left(X_{i}-\mu_{X}\right)\left(Y_{i}-\mu_{Y}\right)}{\sigma_{X}\sigma_{Y}}$ denotes Pearson's correlation coefficient. The ACF coefficients describe the linear correlation between process values at two different time steps *X*_*n*_ and *X*_*n*+*k*_, without taking into account the effect of the intermediate time steps *X*_*n*+*k*−1_, …, *X*_*n*+1_. However, *X*_*n*_ could be correlated with *X*_*n*+*k*_ directly, independent of the intermediate values, or the correlation between *X*_*n*_ and *X*_*n*+*k*_ could be conveyed via the intermediate values and vanish when conditioned on these intermediates. To distinguish these cases, the PACF performs a multivariate regression of *X*_*n*+*k*_ on all values *X*_*n*+*k*−1_, …, *X*_*n*_ and finally records the conditioned or partial correlation between *X*_*n*_ and *X*_*n*+*k*_, removing the effect of the intermediate values:

(3) $$\begin{array}{r} \varphi_{kk}=C\left(X_{n+k},X_{n}|X_{n+k-1},\ldots ,X_{n+1}\right). \end{array}$$

**Figure 1:**
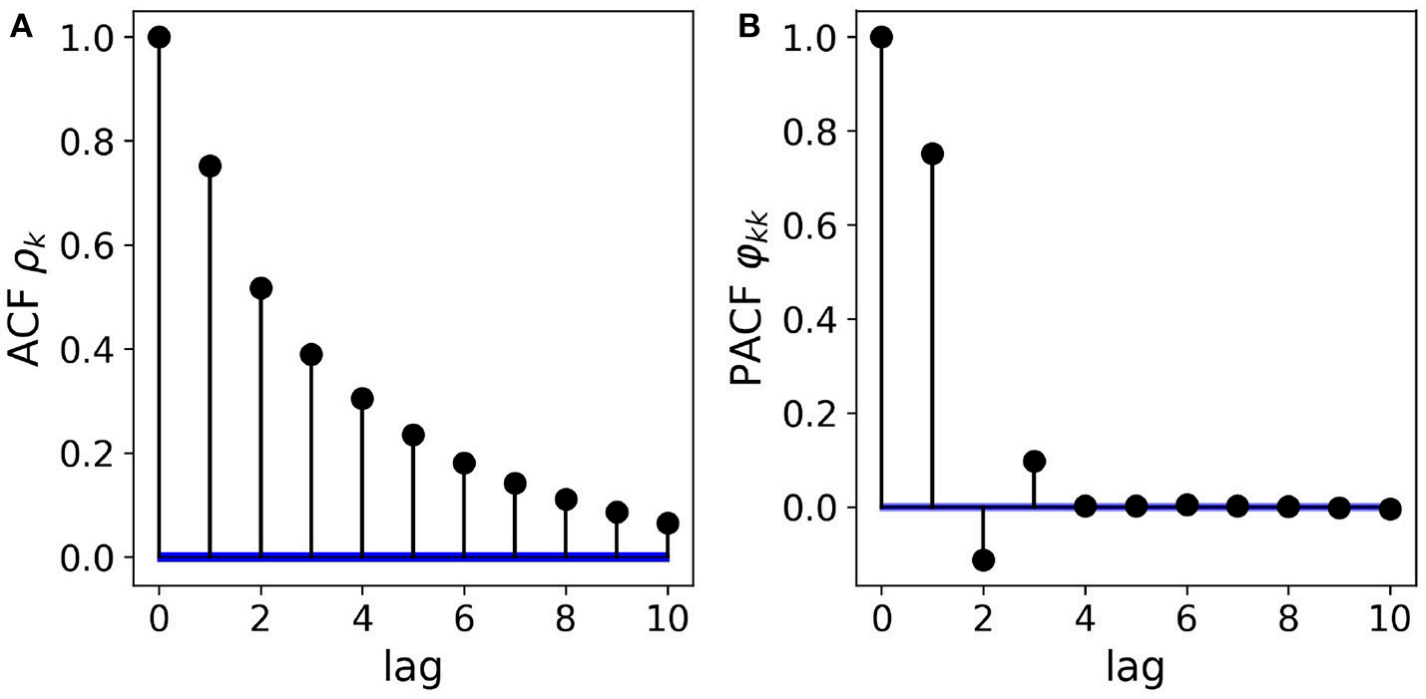
Partial autocorrelation analysis of a real-valued autoregressive process. **(A)** The AR(3) structure of the data cannot be deduced visually from the shape of the ACF, though the exponential decay can be parametrized exactly by the three AR coefficients. **(B)** Removing the effect of intermediate values, the PACF coefficients directly reflect the AR(3) structure as well as the magnitude and sign of the AR coefficients. Confidence intervals (α = 0.05) for the absence of correlations are shown in blue.

Continuous-valued, discrete time AR processes can be systematically assessed using the combination of the autocorrelation function and the PACF (Box and Jenkins, 1976). We here present an example using a third-order autoregressive process that is parametrized by:

(4) $$\begin{array}{rc} X_{n} & =0.85X_{n-1}-0.2X_{n-2}+0.1X_{n-3}+\varepsilon_{n} \end{array}$$

with AR coefficients ϕ_1_ = 0.85, ϕ_2_ = −0.2 and ϕ_3_ = 0.1. Figure 1 shows the ACF/PACF analysis of a simulated sample path (*N* = 10^5^ samples). The left panel, Figure 1A shows the exponentially decaying ACF. Though the analytical form of the ACF can be expressed in terms of the three AR coefficients, visual analysis does not allow to deduce the order of the AR process or the magnitude of the coefficients. The right panel, Figure 1B shows the PACF whose zero-lag coefficient φ_00_ = 1, by definition. The following three values φ_11−33_ directly reflect the relative magnitude and sign of the AR coefficients ϕ_1−3_. Thus, through PACF analysis, the AR(3) structure of the process can be conjectured from visual analysis already. In practice, the statistical significance of each coefficient can also be assessed quantitatively. In Figure 1, confidence intervals (α = 0.05) are shown in blue.

The classical Box-Jenkins approach to time series analysis considers the magnitude of ACF and PACF coefficients to guess the statistical structure of the data (Box and Jenkins, 1976). For a pure autoregressive process of order *p*, the PACF coefficients φ_*kk*_ vanish for *k* > *p*. In case of a pure moving average process, the expected value of the ACF coefficients ρ_*k*_ are zero for *k* > *p*. For mixed (ARMA) processes, the model orders cannot be determined visually. Although the pure AR model order can be deduced from the decay of the PACF, and the PACF coefficients φ_*kk*_ can be expressed in terms of the AR coefficients ϕ_*k*_, the exact value of the AR coefficients ϕ_*k*_ cannot be derived visually, with the exception of a few simple low-order cases.

### 2.2. Information theory

Information theory is rooted in mathematical statistics and uses entropy as one of its main concepts (Kullback, 1959). Entropy characterizes the shape of probability distributions and thereby, the amount of uncertainty or surprise associated with samples generated from the distribution. This section summarizes the concepts and definitions needed to derive the PAIF, more extensive treatments can be found in classical and more recent monographs (Kullback, 1959; Cover and Thomas, 2006; Mézard and Montanari, 2009). Connections of the PAIF with other information-theoretical quantities are derived in the first paragraph of the Results section. Logarithms are computed as log_2_, such that all information-theoretical quantities are measured in bits.

We here consider stochastic processes (_*X*_*n*_)*n*∈ℤ_, i.e., sequences of random variables *X*_*n*_, where each *X*_*n*_ takes values in some finite alphabet of *L* different symbols *S* = {*s*_0_, …, *s*_*L*−1_}. In practice, we deal with finite samples of the underlying process, (_*X*_*n*_)*n* = 0, …, *N*−1_. In the following, contiguous data blocks starting from index *n*, and covering the past *k* values of the process, (*X*_*n*_, *X*_*n*−1_, …*X*_*n*−*k*+1_) will be termed k-histories and are written as

(5) $$\begin{array}{rc} {\text{X}}_{n}^{\left(k\right)} & =\left(X_{n},X_{n-1},\ldots X_{n-k+1}\right). \end{array}$$

Denoting a specific realization of the random variable *X*_*i*_ as *x*_*i*_, the joint probability distribution of k-histories is given by

$$\begin{array}{l} P\left({\text{X}}_{n}^{\left(k\right)}\right)=\text{Pr}\left(X_{n}=x_{n},X_{n-1}=x_{n-1},\ldots X_{n-k+1}=x_{n-k+1}\right). \end{array}$$

where *x*_*i*_ ∈ *S*, for all *i* = *n* − *k* + 1, …, *n*. In the following, the compact notation $P\left({\text{X}}_{n}^{\left(k\right)}\right)$ will be used.

The information content of a random variable *X* with possible values *x*_*i*_ ∈ *S* and associated probabilities *P*(*X* = *x*_*i*_) = *p*_*i*_ is measured by the **Shannon entropy** $H\left(X\right)=-\underset{i}{\sum }p_{i}\log p_{i}$ (Kullback, 1959). The information content of the joint distribution representing the k-history ${\text{X}}_{n}^{\left(k\right)}$ is measured by the **joint entropy**, which is defined as (Kullback, 1959; Cover and Thomas, 2006):

(6) $$\begin{array}{rc} H_{X}\left(n,k\right) & :=H\left({\text{X}}_{n}^{\left(k\right)}\right) \end{array}$$

(7) $$\begin{array}{r} =-\underset{{\text{X}}_{n}^{\left(k\right)}}{\sum }P\left({\text{X}}_{n}^{\left(k\right)}\right)\log P\left({\text{X}}_{n}^{\left(k\right)}\right) \end{array}$$

where the sum runs over all possible values of *X*_*n*_ = *x*_*n*_, …, *X*_*n*−*k*+1_ = *x*_*n*−*k*+1_. The expression *H*_*X*_(*n, k*) contains the time parameter *n*, such that the expression for *H*_*X*_(*n, k*) can be used even in the case of non-stationary processes, whose statistical properties may depend on *n*. Under time-stationary conditions, the entropy is obtained by averaging over all time points and the resulting entropy will be abbreviated

$$H_{k}=\langle H_{X}{\left(n,k\right)}_{n}\rangle$$

where 〈·〉_*n*_ denotes time averaging.

Adding information about the value of another random variable *Y* reduces the uncertainty about *X*, in case *X* and *Y* are statistically dependent. If *X* and *Y* are independent, the entropy of *X* does not change with the additional information about *Y*. To measure the influence of *Y* on *X*, **conditional entropy** is defined as *H*(*X* ∣ *Y*) = *H*(*X, Y*) − *H*(*Y*). In the following, two conditional entropy terms will be used.

The first term is a finite approximation to the entropy rate of the stochastic process *X*. The **entropy rate** *h*_*X*_ of a process quantifies the amount of surprise about the next symbol *X*_*n*+1_ emitted by the process, given knowledge about its past values ${\text{X}}_{n}^{\left(k\right)}$. The theoretical or analytical value *h*_*X*_ is defined via an infinitely long history

$$\begin{array}{ll} h_{X} & =\underset{k\to \infty }{\lim}H\left(X_{n+1}|{\text{X}}_{n}^{\left(k\right)}\right). \end{array}$$

When working with finite experimental data samples, the entropy rate has to be estimated from finite k-histories (Runge et al., 2012; Barnett and Seth, 2015; Faes et al., 2015; Xiong et al., 2017):

(8) $$\begin{array}{r} h_{X}\left(n,k\right)=H\left(X_{n+1}|{\text{X}}_{n}^{\left(k\right)}\right). \end{array}$$

Using the definition of conditional entropy, *h*_*X*_(*n, k*) can be computed from joint entropies as:

(9) $$\begin{array}{r} h_{X}\left(n,k\right)=H\left(X_{n+1},{\text{X}}_{n}^{\left(k\right)}\right)-H\left({\text{X}}_{n}^{\left(k\right)}\right) \end{array}$$

(10) $$\begin{array}{r} =H\left({\text{X}}_{n+1}^{\left(k+1\right)}\right)-H\left({\text{X}}_{n}^{\left(k\right)}\right). \end{array}$$

Following the notation used for Shannon entropy, the time-stationary expression for the entropy rate will be denoted *h*_*k*_ = 〈 *h*_*X*_(*n, k*)〉_*n*_.

The second conditional entropy term used is the **two-point conditional entropy** *H*(*X*_*n*+*k*_ ∣ *X*_*n*_), that measures the amount of information about *X*_*n*+*k*_ contained in *X*_*n*_.

Next, **mutual information** between two random variables is defined as *I*(*X*; *Y*) = *H*(*X*) − *H*(*X* ∣ *Y*) and measures the information shared between both variables. Mutual information will be used to compute two quantities that are useful in characterizing symbol sequences.

First, **active information storage (AIS)** (Lizier et al., 2012) is complementary to the entropy rate. While the entropy rate measures how much information (or surprise) is contained in *X*_*n*+1_, despite knowledge of its k-history ${\text{X}}_{n}^{\left(k\right)}$, AIS measures the amount of common (or shared) information between *X*_*n*+1_ and its k-history. The active information storage term for a history of length k is defined as

(11) $$\begin{array}{r} a_{X}\left(n,k\right)=I\left(X_{n+1};{\text{X}}_{n}^{\left(k\right)}\right) \end{array}$$

and the stationary expression is *a*_*k*_ = < *a*_*X*_(*n, k*)>_*n*_.

For computational implementation, active information storage is decomposed into joint entropy terms:

$$\begin{array}{l} I(X_{n+1};X_{n}^{\left(k\right)})=H(X_{n+1})-H(X_{n+1}|X_{n}^{\left(k\right)})\\ \;=H(X_{n+1})+H(X_{n}^{\left(k\right)})-H(X_{n+1},Xn(k)\\ \;=H(X_{n+1})+H(X_{n}^{\left(k\right)})-H(X_{n+1}^{\left(k+1\right)}). \end{array}$$

The second mutual information term used is *I*(*X*_*n*+1_; *X*_*k*_), and yields an estimate of the statistical dependency between the random variables *X*_*n*_ and *X*_*n*+*k*_. In a recent publication, we used the term **autoinformation function (AIF)** to denote the set of time-lagged mutual information terms computed for a number of time lags (von Wegner et al., 2017). The name AIF was derived from the formal analogy with the autocorrelation function (ACF) for metric time series. We defined the AIF coefficient at time lag *k* as:

(12) $$\begin{array}{rc} \alpha_{X}\left(n,k\right) & =I\left(X_{n+k};X_{n}\right) \end{array}$$

(13) $$\begin{array}{r} =H\left(X_{n+k}\right)-H\left(X_{n+k}|X_{n}\right) \end{array}$$

(14) $$\begin{array}{r} =H\left(X_{n+k}\right)+H\left(X_{n}\right)-H\left(X_{n+k},X_{n}\right) \end{array}$$

and the stationary term is α_*k*_ = < α_*X*_(*n, k*)>_*n*_. Rather than using linear correlation to measure the dependency between two time points, as the ACF does, the AIF employs mutual information between the random variables at time points *n* and *n*+*k*. The measure is symmetric, i.e., *I*(*X*_*n*_; *X*_*n*+*k*_) = *I*(*X*_*n*+*k*_; *X*_*n*_) and therefore does not contain directional information. In analogy to the autocorrelation function, division of all coefficients by α_*X*_(*n*, 0) normalizes the AIF to α_*X*_(*n*, 0) = 1. The computational cost is independent of the time lag *k*, as all entropies are computed from one-dimensional (*H*(*X*_*n*+*k*_), *H*(*X*_*n*_)) and two-dimensional (*H*(*X*_*n*+*k*_, *X*_*n*_)) distributions.

Finally, the definition of partial autoinformation, the central concept of this work, is based on the concept of **conditional mutual information** which includes a third random variable *Z*, on which the mutual information between *X* and *Y* is conditioned:

(15) $$\begin{array}{r} I\left(X;Y|Z\right)=H\left(X|Z\right)-H\left(X|Y,Z\right). \end{array}$$

The information-theoretical match for the PACF should estimate the two-point dependency between *X*_*n*_ and *X*_*n*+*k*_, while removing the influence of the intermediate variables ${\text{X}}_{n+k-1}^{\left(k-1\right)}=\left(X_{n+k-1},\ldots ,X_{n+1}\right)$. This is achieved by computing the conditional mutual information (Equation 15) between *X*_*n*_ and *X*_*n*+*k*_, given ${\text{X}}_{n+k-1}^{\left(k-1\right)}$.

We therefore define the PAIF coefficient π_*X*_(*n, k*) at time lag *k* as:

(16) $$\begin{array}{r} \pi_{X}\left(n,k\right)=I\left(X_{n+k};X_{n}|X_{n+k-1}\ldots X_{n+1}\right) \end{array}$$

(17) $$\begin{array}{r} =I\left(X_{n+k};X_{n}|{\text{X}}_{n+k-1}^{\left(k-1\right)}\right). \end{array}$$

Using the definition of conditional mutual information in terms of conditional entropies, and the expression of conditional entropy in terms of joint entropies, the computation of π_*X*_(*n, k*) can be reduced to the estimation of joint entropies:

(18) $$\begin{array}{r} \pi_{X}\left(n,k\right)=H\left(X_{n+k}|{\text{X}}_{n+k-1}^{\left(k-1\right)}\right)-H\left(X_{n+k}|{\text{X}}_{n+k-1}^{\left(k-1\right)},X_{n}\right)\\ =H\left(X_{n+k},{\text{X}}_{n+k-1}^{\left(k-1\right)}\right)-H\left({\text{X}}_{n+k-1}^{\left(k-1\right)}\right)-H\left(X_{n+k},{\text{X}}_{n+k-1}^{\left(k\right)}\right) \end{array}$$

(19) $$\begin{array}{r} +H\left({\text{X}}_{n+k-1}^{\left(k\right)}\right) \end{array}$$

(20) $$\begin{array}{r} =H\left({\text{X}}_{n+k}^{\left(k\right)}\right)-H\left({\text{X}}_{n+k-1}^{\left(k-1\right)}\right)-H\left({\text{X}}_{n+k}^{\left(k+1\right)}\right)+H\left({\text{X}}_{n+k-1}^{\left(k\right)}\right). \end{array}$$

The stationary expression is

$$\begin{array}{l} \pi_{k}=h_{k-1}-h_{k}\\ \;=-H_{k-1}+2H_{k}-H_{k+1}. \end{array}$$

For the first two coefficients π_*X*_(*n*, 0) and π_*X*_(*n*, 1), there are no intermediate values ${\text{X}}_{n+k-1}^{\left(k-1\right)}$ to condition on. Analogous to the PACF algorithm, we set π_*X*_(*n*, 0) = α_*X*_(*n*, 0) and π_*X*_(*n*, 1) = α_*X*_(*n*, 1). The computational load increases exponentially with history length *k*, as the discrete joint distribution $P\left({\text{X}}_{n+k-1}^{\left(k-1\right)}\right)$ over *L* labels has *L*^*k*−1^ elements.

**Figure 2:**
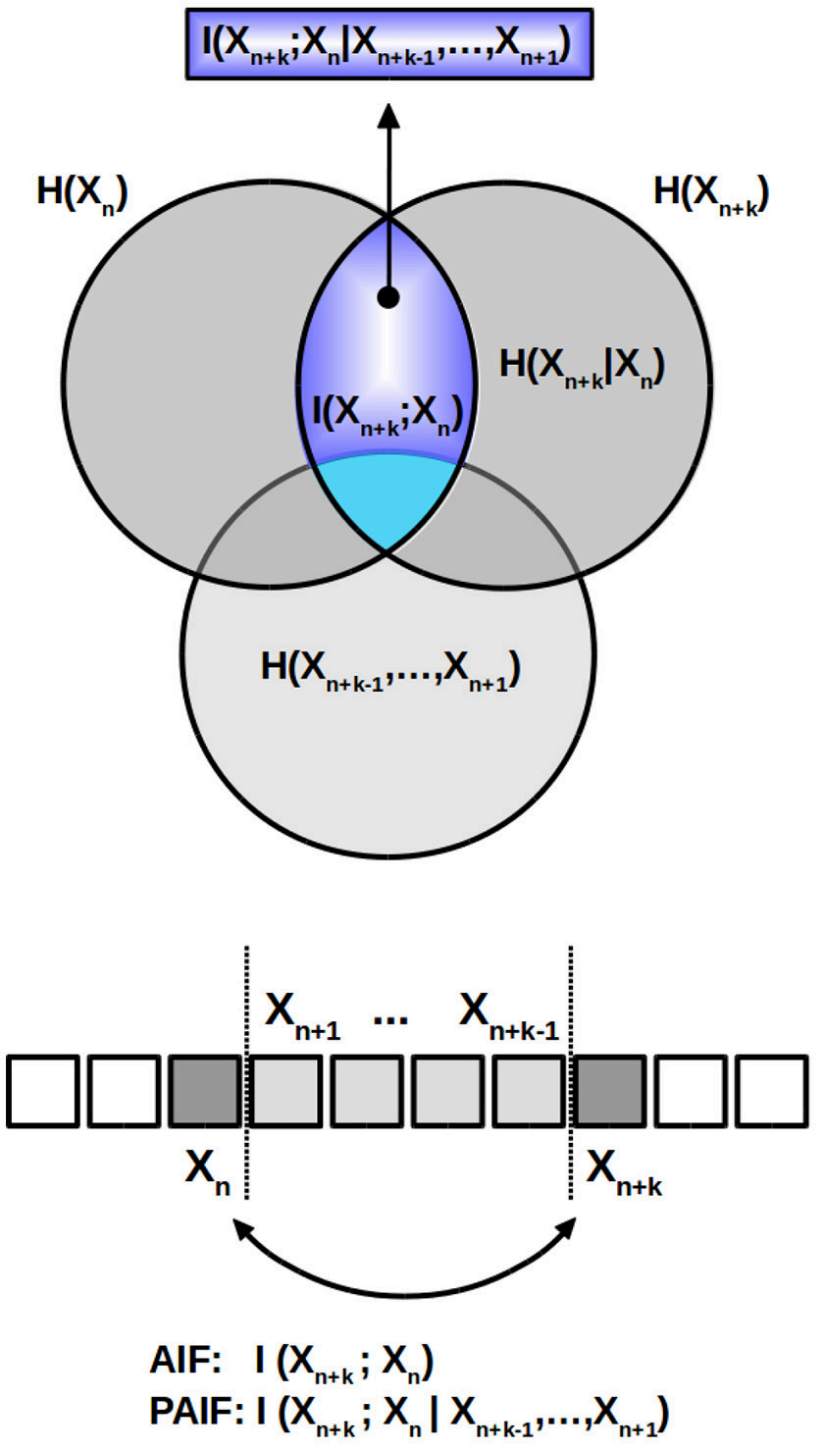
AIF/PAIF analysis. The information diagram above illustrates the partition of the total data entropy. The intersection of the two dark gray circles, representing *H*_*n*_ and *H*_*n*+*k*_, respectively, corresponds to the AIF coefficient *I*(*X*_*n*+*k*_; *X*_*n*_) (light blue area). It measures the shared information between the time points *n* and *n*+*k*, while ignoring the intermediate variables. The PAIF coefficients are represented by the dark blue sub-area of *I*(*X*_*n*+*k*_; *X*_*n*_) that results from excluding all elements that belong to the intermediate values *H*(*X*_*n*+*k*−1_, …, *X*_*n*+1_), shown in light gray color in the Venn diagram and the symbolic time series below.

The computation of the quantity of interest, the PAIF coefficients, is visualized in Figure 2. Above, the relationships between the quantities discussed here are shown as an information diagram, a special form of a Venn diagram. AIF coefficients are represented by the intersection of the two dark gray circles which represent *H*(*X*_*n*+*k*_) and *H*(*X*_*n*_), respectively. In the scheme below, where each element of the time series *X*_*n*_ is visualized as a square box, the AIF coefficients represent the shared information between *X*_*n*+*k*_ and *X*_*n*_, without taking into account the effects of ${\text{X}}_{n+k-1}^{\left(k-1\right)}$ (light gray areas in the information diagram above and the symbolic sequence below). The PAIF corresponds to the part of *I*(*X*_*n*+*k*_; *X*_*n*_) that does not intersect with the lower circle, representing the intermediate values $H\left({\text{X}}_{n+k-1}^{\left(k-1\right)}\right)$. The area that represents the PAIF is shown in dark blue in the information scheme.

### 2.3. Markovianity tests

A discrete Markov process (_*X*_*n*_)*n*∈ℤ_ of order *M* is defined via the property

(21) $$\begin{array}{rc} P\left(X_{n+1}|{\text{X}}_{n}^{\left(M+k\right)}\right) & =P\left(X_{n+1}|{\text{X}}_{n}^{\left(M\right)}\right) \end{array}$$

for all positive integers *k* ≥ 0. In words, the transition probabilities from *X*_*n*_ to the state *X*_*n*+1_ depend on the *M*-history of *X*_*n*_, whereas inclusion of more values from the process' past, beyond *X*_*n*−*M*+1_, does not convey further information about the transition probabilities.

General tests for the Markov property of low orders have been introduced in the 1950s and further tests for many special cases are still being developed today. Early works used analytical expressions for the distribution of symbol counts, given a certain Markov structure, and developed likelihood ratio tests for the cases of known (Bartlett, 1951) and unknown (Hoel, 1954) transition probabilities. Further developments included χ^2^ tests for hypotheses about the time-stationarity of transition probabilities, direct comparisons of different Markov orders (Anderson and Goodman, 1957; Goodman, 1958), as well as parameter estimation methods and tests for continuous time Markov processes (Billingsley, 1961). Using close relationships between χ^2^ statistics and information-theoretical expressions, test statistics based on Kullback-Leibler metrics were summarized as a monograph and in a practice-oriented article containing many numerical examples by Kullback (Kullback, 1959; Kullback et al., 1962). Further approaches include the application of the Akaike information criterion to optimize the order estimate for a discrete Markov chain (Tong, 1975) as well as data compression oriented algorithms (Merhav et al., 1989) and extensive surrogate data tests (Pethel and Hahs, 2014). The results of the PAIF method developed here is compared to the Markov order test presented in van der Heyden et al. (1998). The latter test compares finite entropy rate estimates (*h*_*X*_(*n, k*)) of the data to be tested with surrogate statistics obtained from *M*-order Markov surrogates with the same transition probabilities $P\left(X_{n+1}|{\text{X}}_{n}^{\left(M\right)}\right)$ as the data. The algorithm for the computation of the surrogates is given in detail in (van der Heyden et al., 1998) and is summarized in the following section 2.4. This test will be termed conditional entropy test. In this article, surrogate statistics for each test data set are computed from *n* = 100 surrogate sequences for each Markov order *M* = 0, …, 5. The Markov order identified by the conditional entropy test is taken to be the value *M* for which all *h*_*X*_(*n, k*) lie within the α = 0.05 confidence interval defined by the surrogates.

We recently published our Python implementation of the Markovianity tests of order 0-2 as well as symmetry and stationarity tests as given in Kullback et al. (1962), in article form (von Wegner and Laufs, 2018), and as open-source code. Although the code is part of an algorithm to process EEG microstate sequences, the tests can be exported and applied generically.

### 2.4. Markov surrogate data

A Markov process of order *M* is also defined via its transition probabilities $P\left(X_{n+1}|{\text{X}}_{n}^{\left(M\right)}\right)$, where the probability to go into state *X*_*n*+1_ is conditioned on the *M*-history ${\text{X}}_{n}^{\left(M\right)}$. To synthesize a Markov process of order *M*, using the same transition probabilities as the underlying experimental time series (*X*_*n*_), the empirical *M*-order transition matrix is estimated first. To this end, all contiguous tuples of length (*M*+1) taken from the time series, i.e., tuples of the form (*X*_*n*−*M*+1_, …, *X*_*n*_, *X*_*n*+1_) are considered. The maximum likelihood estimate for the transition probability $P\left(X_{n+1}|{\text{X}}_{n}^{\left(M\right)}\right)$ based on this sample is given by

$$\begin{array}{ll} {\hat{p}}_{ML}\left(X_{n+1}|{\text{X}}_{n}^{\left(M\right)}\right) & =\frac{\#\left(X_{n+1},{\text{X}}_{n}^{\left(M\right)}\right)}{\#\left({\text{X}}_{n}^{\left(M\right)}\right)} \end{array}$$

where #(·) denotes the number of times a specific outcome occurs in the empirical sequence (*X*_*n*_) (Anderson and Goodman, 1957; van der Heyden et al., 1998). For instance, $\#\left({\text{X}}_{n}^{\left(M\right)}\right)$ is the number of realizations (*X*_*n*_ = *x*_*n*_, …, *X*_*n*−*M*+1_ = *x*_*n*−*M*+1_). While counting the tuples, the joint distribution of ${\text{X}}_{n}^{\left(M\right)}$ is recorded at the same time.

Following van der Heyden et al. (1998), the first *M* values of each surrogate Markov sequence are initialized with a sample from the joint distribution ${\text{X}}_{n}^{\left(M\right)}$. From there, we have a *M*-history ${\text{X}}_{n}^{\left(M\right)}$ for every subsequent value *X*_*n*+1_. The value of *X*_*n*+1_ is chosen according to the transition probabilities ${\hat{p}}_{ML}\left(X_{n+1}|{\text{X}}_{n}^{\left(M\right)}\right)$ and the given *M*-history. Given a specific *M*-history ${\text{X}}_{n}^{\left(M\right)}$, there are *L* transition probabilities *q*_0_, …, *q*_*L*−1_, where $q_{i}={\hat{p}}_{ML}\left(X_{n+1}=s_{i}|{\text{X}}_{n}^{\left(M\right)}\right)$. The distribution of the state *X*_*n*+1_ = *s*_*i*_ is sampled correctly using a pseudo-random number *r*, uniformly distributed on the unit interval, $r\sim U_{\left[0,1\right]}$, and the condition $\overset{i-1}{\underset{l=0}{\sum }}q_{l}\leq r<\overset{i}{\underset{l=0}{\sum }}$.

We recently published a Python implementation for first-order Markov surrogates in the open-source package described in von Wegner and Laufs (2018), and have included the *M*-order Markov surrogates in the Github repository associated with this paper.

### 2.5. The two-state Markov process

The general concepts introduced above are easily applied to a two-state, first-order Markov process that can be written as

$$\begin{array}{l} A\overset{p}{\underset{q}{⇌}}B \end{array}$$

with transition rates *p* and *q*. The self-transition rate for *A*→*A* is 1−*p*, and the rate of *B*→*B* is 1−*q*. The complete transition matrix *T* reads

$$T=\left(\begin{array}{cc} 1-p & p\\ q & 1-q \end{array}\right)$$

and has eigenvalues λ_0_ = 1 and λ_1_ = 1 − (*p* + *q*). The eigenvalue λ_0_ = 1 is assured by the Perron-Frobenius theorem as *T* is a stochastic matrix, i.e., $\underset{j}{\sum }T_{ij}=1$ for all *i*. The normalized positive eigenvector to λ_0_ is the equilibrium or stationary distribution *p*_st_ of the process,

$$\begin{array}{l} p_{\text{st}}=\left(\frac{q}{p+q},\frac{p}{p+q}\right). \end{array}$$

We set $p_{A}=\frac{q}{p+q}$ and $p_{B}=\frac{p}{p+q}$. With the auxiliary functions φ, ψ : [0, 1] → ℝ defined as φ(*x*) = −*x*log*x* and *ψ*(*x*) = φ(*x*)+φ(1−*x*), the analytical quantities *H*_*pq*_, *h*_*pq*_ and *a*_*pq*_ for the 2-state first-order Markov process acquire a very simple form.

The Shannon entropy of the 2-state Markov process is

$$\begin{array}{l} H_{pq}=-p_{A}\log p_{A}-p_{B}\log p_{B}\\ \;=\varphi (p_{A})+\varphi (1-p_{A})\\ \;=\psi (p_{A}). \end{array}$$

Due to the Markov property, the entropy rate is *h*_*pq*_ = *H*(*X*_*n*+1_ ∣ *X*_*n*_) and evaluates to

$$\begin{array}{l} h_{pq}=-p_{A}\left[(1-p)\log(1-p)+p\log p\right]-p_{B}[q\log q\\ \;+(1-q)\log(1-q)]=p_{A}\psi (p)+p_{B}\psi (q). \end{array}$$

The Markov property reduces the full expression for information storage $I\left(X_{n+1};{\text{X}}_{n}^{\left(k\right)}\right)$ to *a*_*pq*_ = *I*(*X*_*n*+1_; *X*_*n*_):

$$\begin{array}{l} a_{pq}=-p_{A}\log p_{A}-p_{B}\log p_{B}\\ \;+p_{A}\left[(1-p)\log(1-p)+p\log p\right]+p_{B}\left[q\log q+(1-q)\log(1-q)\right]\\ \;=\psi (p_{A})-p_{A}\psi (p)-p_{B}\psi (q). \end{array}$$

The total entropy is conserved between active information storage and the entropy rate:

(22) $$\begin{array}{r} H_{pq}=a_{pq}+h_{pq}. \end{array}$$

To validate the proposed approach, these analytical results will later be compared with numerical results of a hidden Markov process classified as first-order Markovian by the PAIF method.

### 2.6. Higher-order Markov processes

To test the properties of the PAIF, two higher-order Markov processes with known properties are synthesized.

The first process is a third-order Markov process denoted *MC*_1..*M* = 3_, with transition probabilities that depend on ${\text{X}}_{n}^{\left(M=3\right)}$. Given *L* states, there are *L*^*M*^ possible *M*-histories preceding *X*_*n*+1_, such that $P\left(X_{n+1}|{\text{X}}_{n}^{\left(M\right)}\right)$ in matrix form has shape (*L*^*M*^, *L*). The specific transition probabilities are random numbers fulfilling $\underset{j}{\sum }P\left(X_{n+1}=s_{j}|{\text{X}}_{n}^{\left(M\right)}\right)=1$ for all *M*-histories ${\text{X}}_{n}^{\left(M\right)}$. Sample paths are generated using the method described in section 2.4.

The second process will be termed *MC*_*M* = 3_ and is constructed in such a way that the *X*_*n*_ → *X*_*n*+1_ transition only depends on *X*_*n*−2_. Like the first process, this process can also be classified as third-order Markovian (*M* = 3), with the particular property that the influence of *X*_*n*_ and *X*_*n*−1_ vanishes.

### 2.7. Hidden Markov processes

A more general class of discrete processes is represented by probabilistic finite state machines (Crutchfield and Young, 1989), which implement hidden Markov models (HMMs). Hidden Markov models generate sequences of symbols defined over a set of observable states that correspond to our measurements. The observable symbols are emitted by a set of hidden states that follow a Markov process, usually of first order. Each hidden state emits the observable symbols according to its own probability distribution defined over the observable set. It is important to note that the sequence of emitted symbols does not necessarily follow a Markov law.

#### 2.7.1. Even process

The even process is a non-Markov process with two hidden states ({*A, B*}) and two observables ({0, 1}). The process scheme is visualized in Figure 4A. The process can emit arbitrarily long sequences of zeros by repeated self-transitions of the hidden state *A* → *A*. With probability *p* = 0.5, the state *A* can switch to *B* and hereby emit a 1, which is followed by another 1 with probability *p* = 1. Thus, ones are always generated in pairs, i.e., in blocks of even length. The procedure generates dependencies that in theory reach into the infinite past and can therefore not be reduced to a Markov process.

#### 2.7.2. Golden mean process

Two different implementations of the Golden-mean process are used. First, a 2-state first-order Markovian implementation using two hidden states ({*A, B*}) and two observable states ({0, 1}) (Ara et al., 2016), and second, a fourth-order Markov implementation using seven hidden ({*A*−*G*}) and two observable states ({0, 1}) (Mahoney et al., 2016). The scheme of the 2-state process (Figure 4B) is structurally similar to the even process, but dynamically different. Ones are never emitted repeatedly, i.e., they are always preceded and followed by a zero, in contrast to the even process. The 7-state golden mean process is a so-called *R, k*-Markov process with Markov order *R* = 4 and cryptic order *k* = 3, in our case (Mahoney et al., 2016).

### 2.8. Ising model data

The Ising model is a widely used discrete model from statistical physics (Hohenberg and Halperin, 1977). The model describes the ferromagnetic interaction of elementary spin variables, with two possible values ±1, as a function of temperature and the coupling coefficients between spins. The model can be realized with different geometries and in many cases, shows a phase transition at a critical temperature. We use a 2D square lattice geometry (*L* = 50) and run the system for 10^6^ time steps. Sample paths are generated by Monte Carlo simulation using a standard Gibbs sampling scheme (Bortz et al., 1975).

### 2.9. Simulated ion channel data

The dynamics of a simple ion channel with one open and one closed state is modeled as a motion of a particle in the double-well potential $V\left(x\right)=-\frac{a}{2}x^{2}+\frac{b}{4}x^{4}$, which shows two stable local minima at $x_{1,2}=\pm \sqrt{\frac{a}{b}}$ and one unstable local maximum at *x*_0_ = 0 (Liebovitch and Czegledy, 1992; von Wegner et al., 2014). The system is excited by thermal noise, as implemented by iid Gaussian pseudo-random numbers ξ_*n*_. The system is described by von Wegner et al. (2014)

(23) $$\begin{array}{r} X_{n+1}=X_{n}+\left(aX_{n}-bX_{n}^{3}\right)dt+\xi_{n} \end{array}$$

and integrated with an Euler scheme and dt = 10^−3^.

### 2.10. EEG microstate sequences

A resting state EEG data set from a 21 year old, healthy right-handed female during wakeful rest was selected and analyzed. The data set is part of a larger database for which we have reported the detailed pre-processing pipeline before (von Wegner et al., 2016, 2017). The 30 channel EEG raw data was sampled at 5 kHz using the standard 10−10 electrode configuration, band-pass filtered to the 1−30 Hz range using a zero-phase Butterworth filter with a slope of 24 dB/octave, down-sampled to 250 Hz and re-referenced to an average reference. Written informed consent was obtained from the subject and the study was approved by the ethics committee of the Goethe University, Frankfurt, Germany. EEG microstates were identified using the first four principal components (PCA analysis) of the data set and the symbolic microstate sequence was obtained by competitively fitting the microstate maps back into the EEG data set as detailed in (von Wegner et al., 2016, 2017).

## 3. Results

### 3.1. Theoretical results: relations between measures

Using the time index *n* + *k* as a reference, the partial autoinformation coefficients $\pi_{X}\left(n,k\right)=I\left(X_{n+k};X_{n}|{\text{X}}_{n+k-1}^{\left(k-1\right)}\right)$ can be related to the entropy rate $h_{X}\left(n+k-1,k\right)=H\left(X_{n+k}|{\text{X}}_{n+k-1}^{\left(k\right)}\right)$ and to active information storage $a_{X}\left(n+k-1,k\right)=I\left(X_{n+k};{\text{X}}_{n+k-1}^{\left(k\right)}\right)$ as follows.

The entropy rate *h*_*X*_(*n* + *k* − 1, *k*) can be written as the difference of two joint entropies of different lengths (Equation 9), $h_{X}\left(n+k-1,k\right)=H\left({\text{X}}_{n+k}^{\left(k+1\right)}\right)-H\left({\text{X}}_{n+k-1}^{\left(k\right)}\right)$.

Next, active information storage can be expressed as the difference of a joint entropy and the entropy rate:

$$\begin{array}{l} a_{X}(n+k-1,k)=I(X_{n+k};X_{n+k-1}^{\left(k\right)})\\ \;=H(X_{n+k})-H(X_{n+k}|X_{n+k-1}^{\left(k\right)})\\ \;=H(X_{n+k})-h_{X}(n+k-1,k). \end{array}$$

In the stationary case, we have

(24) $$\begin{array}{r} H_{1}=a_{k}+h_{k}. \end{array}$$

Similar to the case presented for the 2-state Markov process, it is observed that also in the general case, the entropy *H*(*X*_*n*+*k*_) is conserved, being the sum of active information storage and the entropy rate. In words, the information about the future state *X*_*n*+*k*_ is the sum of the actively stored information from time step *n* up to time step *n* + *k* − 1, and the entropy rate between time steps *n* + *k* − 1 to *n* + *k*.

Finally, the PAIF coefficient π_*X*_(*n, k*) can be written as the difference of entropy rates for different history lengths:

$$\begin{array}{l} \pi_{X}\left(n,k\right)=I\left(X_{n+k};X_{n}|{\text{X}}_{n+k-1}^{\left(k-1\right)}\right)\\ =H\left(X_{n+k}|{\text{X}}_{n+k-1}^{\left(k-1\right)}\right)-H\left(X_{n+k}|{\text{X}}_{n+k-1}^{\left(k\right)}\right)\\ =h_{X}\left(n+k-1,k-1\right)-h_{X}\left(n+k-1,k\right). \end{array}$$

Alternatively, π_*X*_(*n, k*) can also be decomposed into a difference of AIS terms for two different history lengths:

$$\begin{array}{l} \pi_{X}(n,k)\;=I(X_{n+k};X_{n}|X_{n+k-1}^{\left(k-1\right)})\\ \;=H(X_{n+k}|X_{n+k-1}^{\left(k-1\right)})-H(X_{n+k}|X_{n+k-1}^{\left(k-1\right)},X_{n})\\ \;=H(X_{n+k}|X_{n+k-1}^{\left(k-1\right)})-H(X_{n+k}|X_{n+k-1}^{\left(k\right)}\\ \;=H(X_{n+k})-H(X_{n+k}|X_{n+k-1}^{\left(k\right)})-H(X_{n+k})\\ \;-H(X_{n+k}|X_{n+k-1}^{\left(k-1\right)})]\\ \;=I(X_{n+k};X_{n+k-1}^{\left(k\right)})-I(X_{n+k};X_{n+k-1}^{\left(k-1\right)})\\ \;{\text{= a}}_{X}\text{(n + k }-\text{ 1, k) }-{\text{ a}}_{X}\text{(n + k }-\text{1, k}-\text{1).} \end{array}$$

Going from line 3 to line 4, we simply added and subtracted *H*(*X*_*n*+*k*_). In words, the PAIF at time lag *k* is the difference between two AIF terms with history lengths *k* and *k*−1, respectively.

The results can be summarized in a more compact form using the stationary expressions:

(25) $$\begin{array}{rc} \pi_{k} & =a_{k}-a_{k-1} \end{array}$$

(26) $$\begin{array}{r} =h_{k-1}-h_{k}. \end{array}$$

For stationary Markov processes, the joint Shannon entropy *H*_*k*_ exists and the *k*-order entropy rate estimates *h*_*k*_ converge in the limit of *k* → ∞ (Cover and Thomas, 2006). Using Equation 24, it follows that the AIS coefficients *a*_*k*_ also converge. Thus, $\underset{k\to \infty }{\lim}a_{k}-a_{k-1}=0$ and $\underset{k\to \infty }{\lim}h_{k-1}-h_{k}=0$. Using Equation 25, we deduce that the PACF coefficients π_*k*_ also vanish in the large *k* limit:

$$\begin{array}{l} \underset{k\to \infty }{\lim}\pi_{k}=0. \end{array}$$

#### 3.1.1. Markovianity

Using the Markov property defined in Equation 21, it is straightforward to prove that for a stationary Markov process of order *M*, the PAIF coefficients vanish (π_*X*_(*n, k*) = 0) for *k* > *M*:

$$\begin{array}{l} \pi_{X}\left(n,k\right)=I\left(X_{n+k};X_{n}|{\text{X}}_{n+k-1}^{\left(k-1\right)}\right)\\ =H\left(X_{n+k}|{\text{X}}_{n+k-1}^{\left(k-1\right)}\right)-H\left(X_{n+k}|{\text{X}}_{n+k-1}^{\left(k\right)}\right)\\ =h_{k-1}-h_{k}\\ =h_{M}-h_{M}\\ =0. \end{array}$$

Let the first- and second-order finite differences of an arbitrary discrete function *f*_*k*_ of integer parameter *k* be defined as δ_*k*_ *f*_*k*_ = *f*_*k*_ − *f*_*k* − 1_, and $\delta_{k}^{2}f_{k}=f_{k+1}-2f_{k}+f_{k-1}$, then we get

$$\begin{array}{l} \pi_{k}=h_{k-1}-h_{k}\\ =-\delta_{k}h_{k}\\ =-\delta_{k}^{2}H_{k}. \end{array}$$

Thus, the Markovianity test proposed in (van der Heyden et al., 1998) addresses a sequence of entropy rates *h*_*k*_, for different history lengths *k*, which is the negative first-order difference of the sequence of Shannon entropies *H*_*k*_. PAIF analysis uses the second-order difference of the sequence of Shannon entropies $\pi_{k}=-\delta_{k}^{2}H_{k}$. The advantage of the PAIF analysis is the visual exploration of the coefficients, that are equal to zero for *k* > *M*, exactly like in visual PACF diagnostics for metric time series.

### 3.2. Higher-order Markov processes

**Figure 3:**
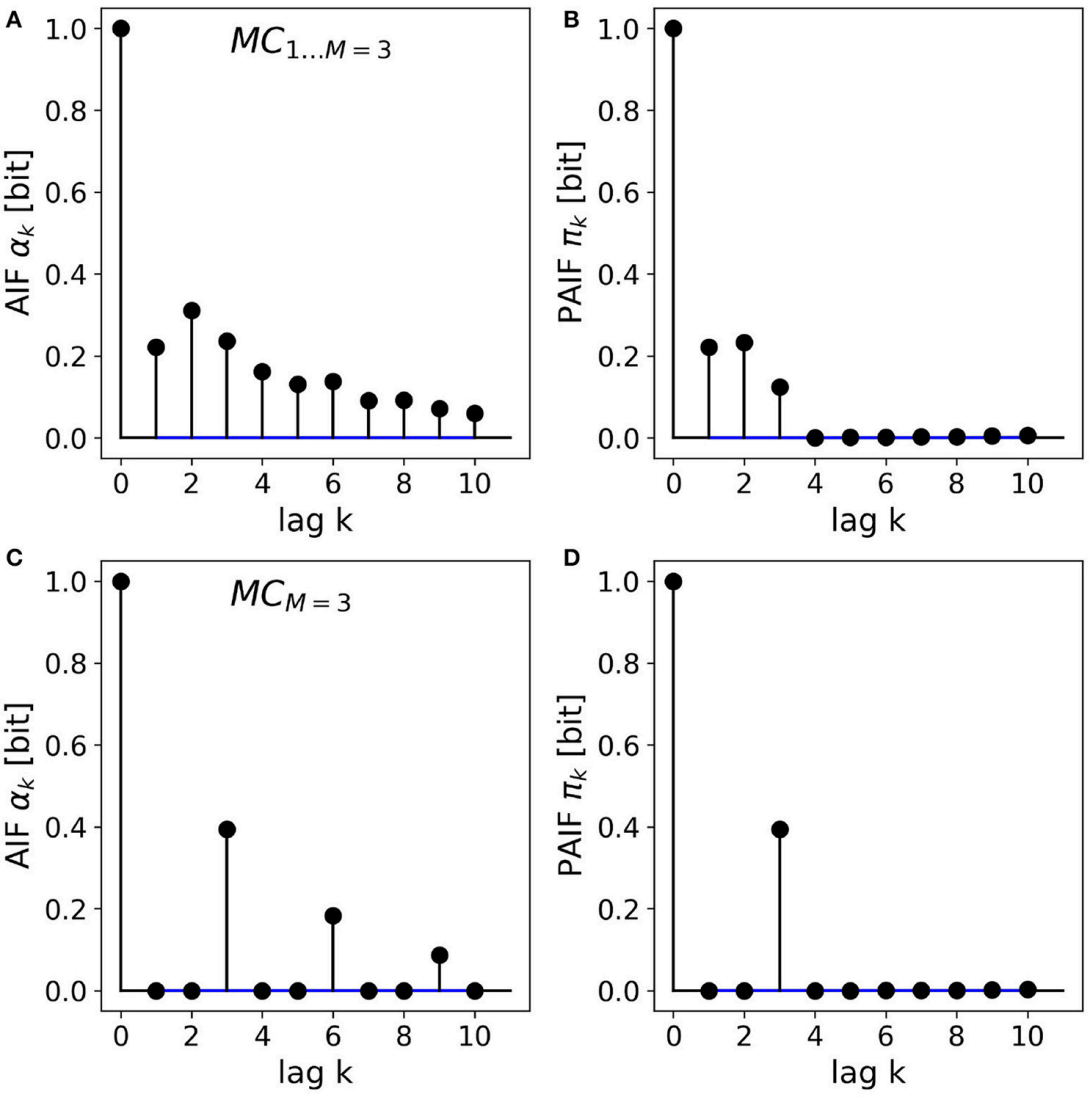
AIF/PAIF analysis of two third-order Markov process samples. **(A)** The AIF of the *MC*_1…*M* = 3_ process slowly decays toward zero and does not reveal the Markov order of the process. **(B)** The PAIF of *MC*_1…*M* = 3_ shows a cutoff after *k* = 3 coefficients, in accordance with the nominal Markov order. **(C)** The AIF of the *MC*_*M* = 3_ process has period 3 and thus hints at the memory structure of the process. **(D)** The PAIF of the *MC*_*M* = 3_ process clearly identifies the Markov order of the process by a distinct peak at the time lag corresponding to the correct model order *M* = *k* = 3.

The results for the two third-order Markov processes are shown in Figures 3A–D. The AIF and PAIF for the third-order Markov processes *MC*_1…*M* = 3_ is shown in Figures 3A,B, respectively. The shape of the AIF does not reveal the third-order dependencies by visual inspection. The PAIF, however, clearly reflects the construction of the process, showing significant PAIF coefficients only up to time lag *k* = 3.

For the second process, *MC*_*M* = 3_, the entropy dynamics can already be estimated by visual inspection of the AIF, which shows a clear periodicity (Figure 3C). Significant PAIF coefficients only occur at time lags that are multiples of the Markov order, *M* = 3. The PAIF (Figure 3D) however, demonstrates the Markov structure of the process in a single significant coefficient π_3_.

Kullback's Markovianity tests of order 0-2 rejected the Markovian null hypotheses for both processes, as expected for Markov processes of order three, by construction. The conditional entropy test correctly identified the Markov order *M* = 3 in both cases.

Confidence intervals (α = 0.05) constructed from uncorrelated surrogate time series are shown in blue. Due to their small magnitude, they visually appear as lines.

### 3.3. Hidden Markov models

**Figure 4:**
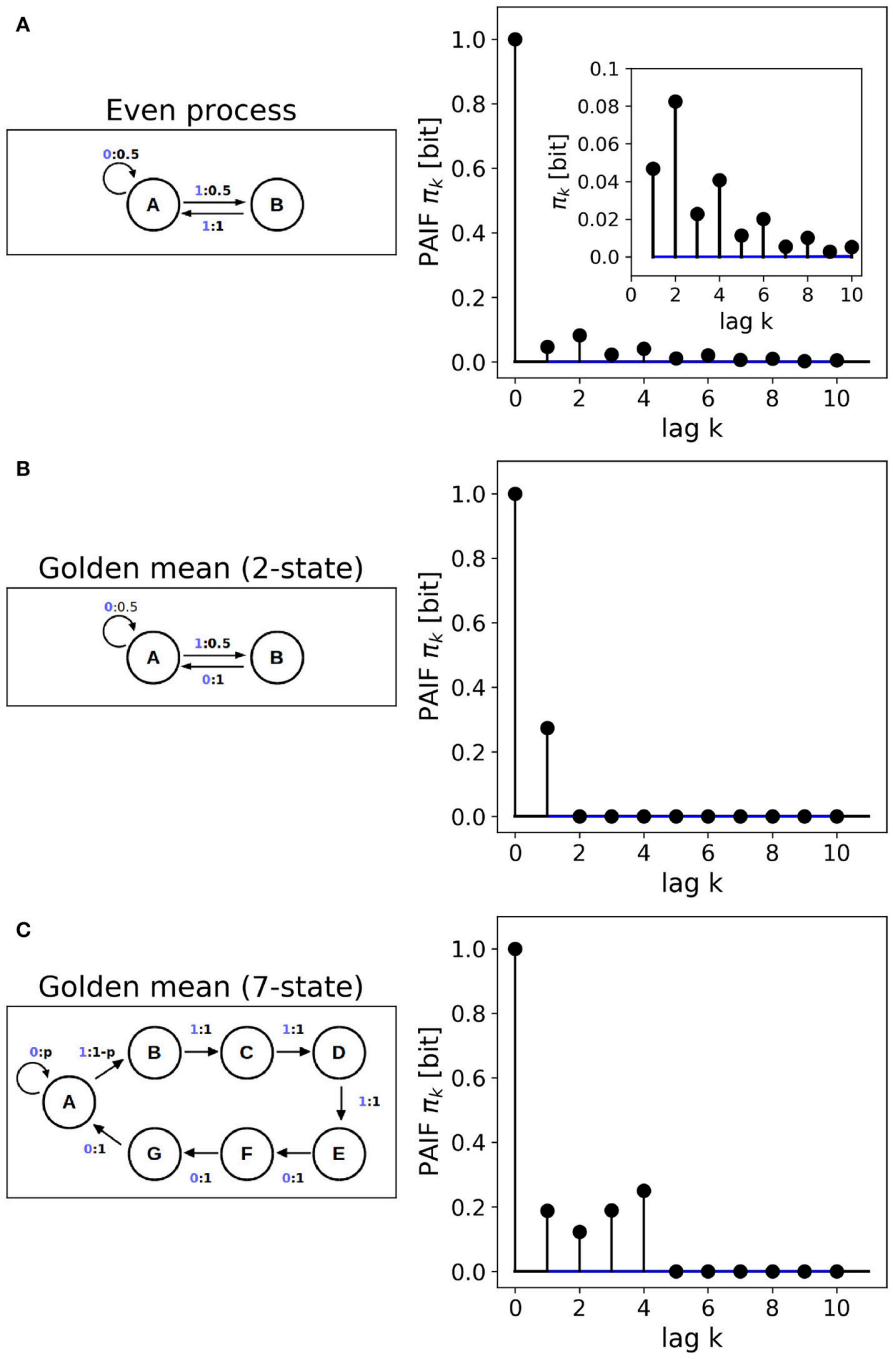
Finite state machines. **(A)** Non-Markovian even process, Markov order *M* = ∞. **(B)** 2-state implementation of the golden-mean process, Markov order *M* = 1. **(C)** 7-state implementation of the golden-mean process, Markov order *M* = 4. The Markov orders are correctly identified by the PAIF approach.

Figure 4 shows the results obtained from HMM data. First, the non-Markovian even process is analyzed. To the right of the HMM scheme, Figure 4A shows the PAIF of a single sample path of length *n* = 10^6^. The inset shows that for all tested time lags the PAIF coefficients lie above the iid confidence interval (blue lines). Thus, PAIF analysis suggests that we are observing a non-Markov process with extended memory effects.

Figure 4B shows the PAIF of the 2-state golden mean process. The PAIF has two significant coefficients π_0_, π_1_ and decays to zero for all other time lags. The PAIF thus classifies the process correctly as a first-order Markov process, despite the hidden Markov implementation. Due to the Markov property, the process can also be represented by a transition matrix and an equilibrium distribution. The associated transition matrix *T* is

$$T=\left(\begin{array}{l} \frac{1}{2}\;\frac{1}{2}\\ 1\;0 \end{array}\right)$$

with stationary distribution $p_{\text{st}}=\left[\frac{2}{3},\frac{1}{3}\right]$. Using these quantities, the theoretical results from Section 2.5 can be applied. Using finite histories (*k* = 2…10), entropy conservation (Equation 24) is fulfilled with a maximum error of 7.25 × 10^−4^, where the error was calculated as $\frac{H_{1}-h_{k}-a_{k}}{H_{1}}$. Based on this analysis, the Shannon entropy of a single symbol is *H*_1_ = 0.919 bit, and consists of an entropy rate of *h*_*X*_ = 0.669 bit and active information storage of *a*_*X*_ = 0.253 bit.

The 7-state HMM of the golden mean process is analyzed in Figure 4C. The fourth-order Markov structure of the implementation is clearly reflected by the PAIF that shows four positive coefficients. The PAIF captures the correct Markov order although the model contains seven hidden states and emits two observable symbols.

Kullback's Markovianity tests of order 0–2 correctly classified the 2-state golden mean process as first-order Markovian. The p-values for orders 0-2 were *p*_0_ = 0.000, *p*_1_ = 0.697, and *p* = 0.990, respectively. For the non-Markovian even process and the fourth-order Markovian 7-state golden mean process, low-order (0-2) Markovianity was correctly rejected. The conditional entropy test correctly identified the Markov properties of all three processes, i.e., found first- and fourth-order properties for the 2-state and 7-state golden mean processes, respectively, and an order *M*>5 for the even process.

### 3.4. The ising model

**Figure 5:**
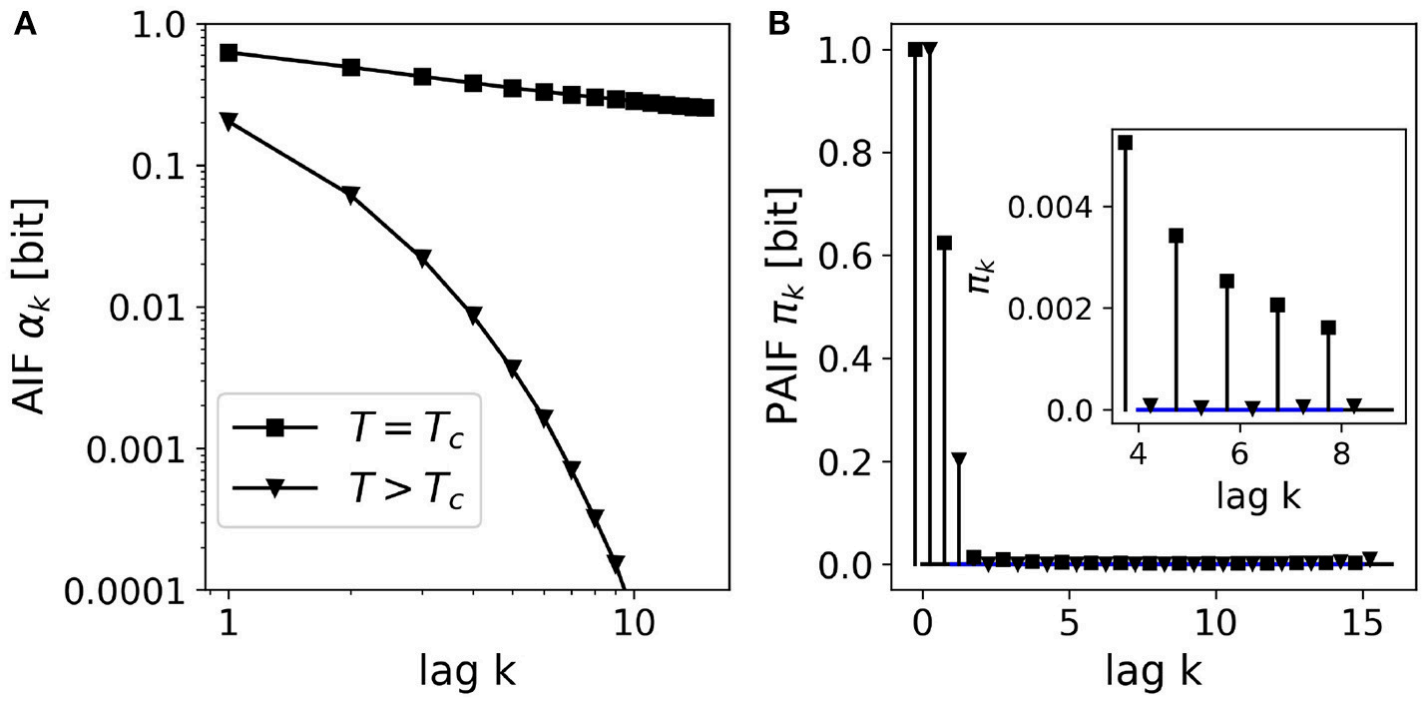
AIF/PAIF analysis of 2D-Ising model data: Results for single lattice site time series (*n* = 10^6^ samples) at two temperatures are shown, close to the critical temperature *T*_*c*_≈2.27 (black squares), and at a higher temperature far from the critical point, *T* = 5.00 (black triangles). **(A)** The AIF is shown in log-log coordinates to better visualize the qualitative difference between the power-law decay (linear in log-log coordinates) at the critical temperature (*T* = *T*_*c*_), and the exponential decay at higher temperatures (*T* = 3.00). **(B)** The PAIF for both temperatures illustrates a dominant coefficient π_1_. However, the inset shows significant positive PAIF coefficients and thus, non-Markovian behavior close at the critical temperature (squares).

We simulated an Ising model on a 2D lattice (50 × 50 elements) at two temperatures, (i) around the critical temperature $T_{c}=\frac{2}{1+\sqrt{2}}\approx 2.27$, and (ii) at a higher temperature *T* = 5.00. From statistical physics, it is known that the system's autocorrelation function shows a slow, power-law decay at the critical point, and an exponential decay far from the critical point where dynamics are dominated by thermal fluctuations. AIF/PAIF analysis was performed on time series of 10^6^ samples of a randomly selected lattice site. The results are shown in Figure 5.

In contrast to the other figures in this manuscript, the AIF in Figure 5A is shown in log-log coordinates, to better visualize the difference between exponential and power-law behavior. The AIF at the critical point *T*_*c*_ shows an almost linear behavior in log-log coordinates (black squares), indicating very slow relaxation dynamics, as expected. For the higher temperature, far from the critical point (*T* = 5.0, black triangles), however, we observe a quickly decaying autoinformation trace, in accordance with results from classical time series analysis. Figure 5B shows the PAIF in linear coordinates, as in all other figures. It is observed that in both cases, *T* = *T*_*c*_, *T* = 5.0, the PAIF profiles seem to be similar. We find two positive PAIF coefficients π_0_, π_1_, and significantly smaller PAIF coefficients for larger time lags. The inset, however, shows that at the critical temperature (squares), the PAIF coefficients lie above the confidence interval, demonstrating non-Markovian, long-range memory effects where the system undergoes a phase transition.

### 3.5. Simulated ion-channel data

**Figure 6:**
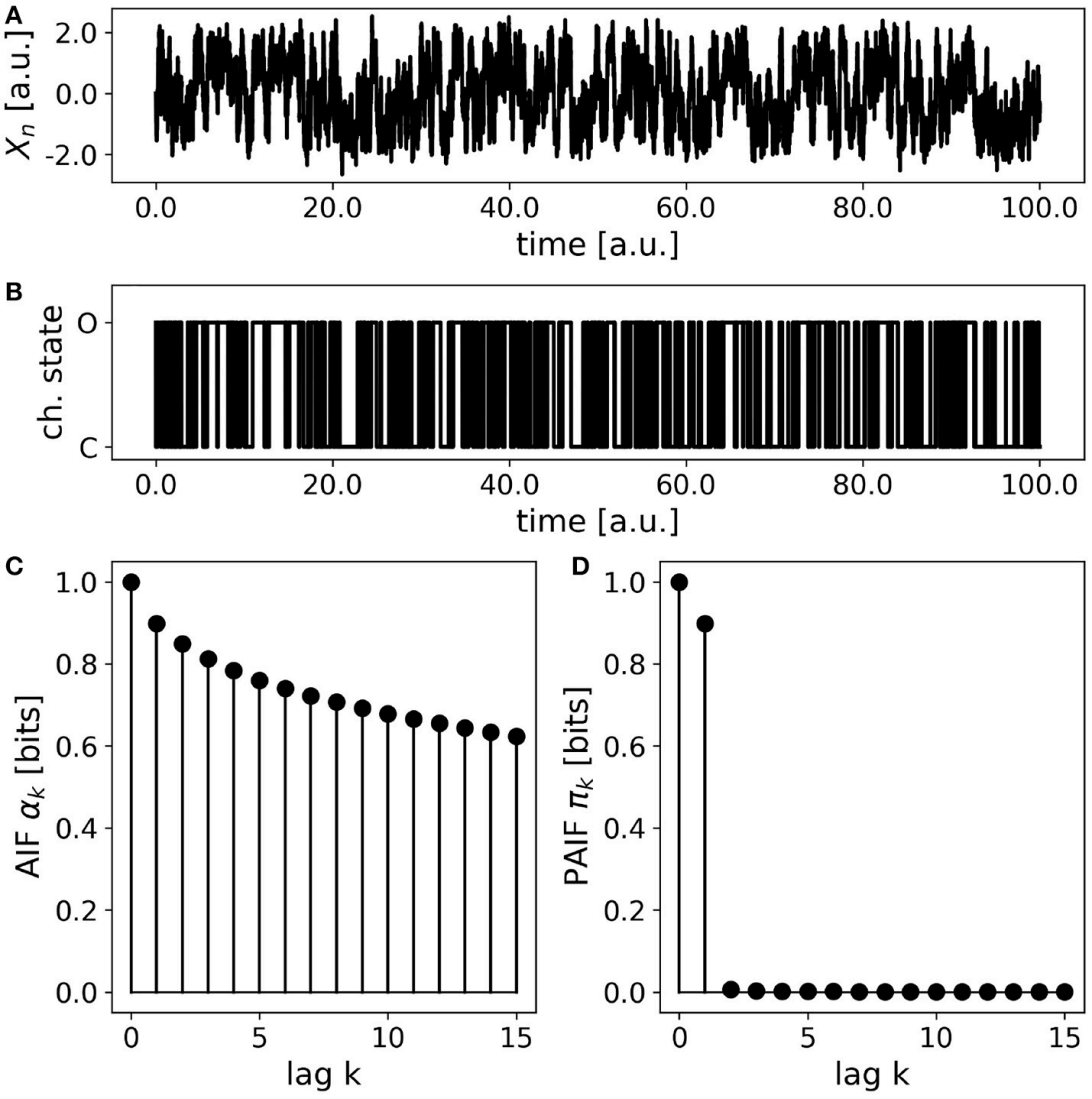
Simulated ion channel data. **(A)** A continuous stochastic process *X*_*n*_ is obtained from a simulation of a double-well potential. A bistable behavior resembling ion channel recordings is observed. **(B)** Thresholding the continuous variable *X*_*n*_ into an open state (O, *X*_*n*_>0) and a close state (C, *X*_*n*_ < 0) yields a symbolic, binary process. **(C)** The AIF of the binary process shows a slow decay without revealing the Markov order of the process. **(D)** The PAIF suggests first-order Markov dynamics by vanishing PAIF coefficients π_*k*_ for *k*>1.

Simplified ion channel dynamics were generated by integration of Equation 23, representing the motion of a particle in a bistable potential, for instance an ion channel with two metastable states corresponding to the open (O) and close (C) state, respectively. To obtain a symbolic time series of O- and C-states, the continuous variable *X*_*n*_ is thresholded at a value of zero. Thereby, all positive values *X*_*n*_>0 are assigned to the open state (O), and all negative values (*X*_*n*_ < 0) are mapped to the close state. The AIF/PAIF analysis of the thresholded signal simulating electrophysiological ion channel data is shown in Figure 6. We observe a slowly decaying AIF (Figure 6A) without any information about the Markov order of the signal. The PAIF profile shows large coefficients π_0_ and π_1_, followed by vanishing PAIF coefficients for *k*>1. Though Markovian dynamics are expected for the continuous dynamics, it is not obvious that the Markov property could be detected after thresholding.

### 3.6. EEG microstate sequences

**Figure 7:**
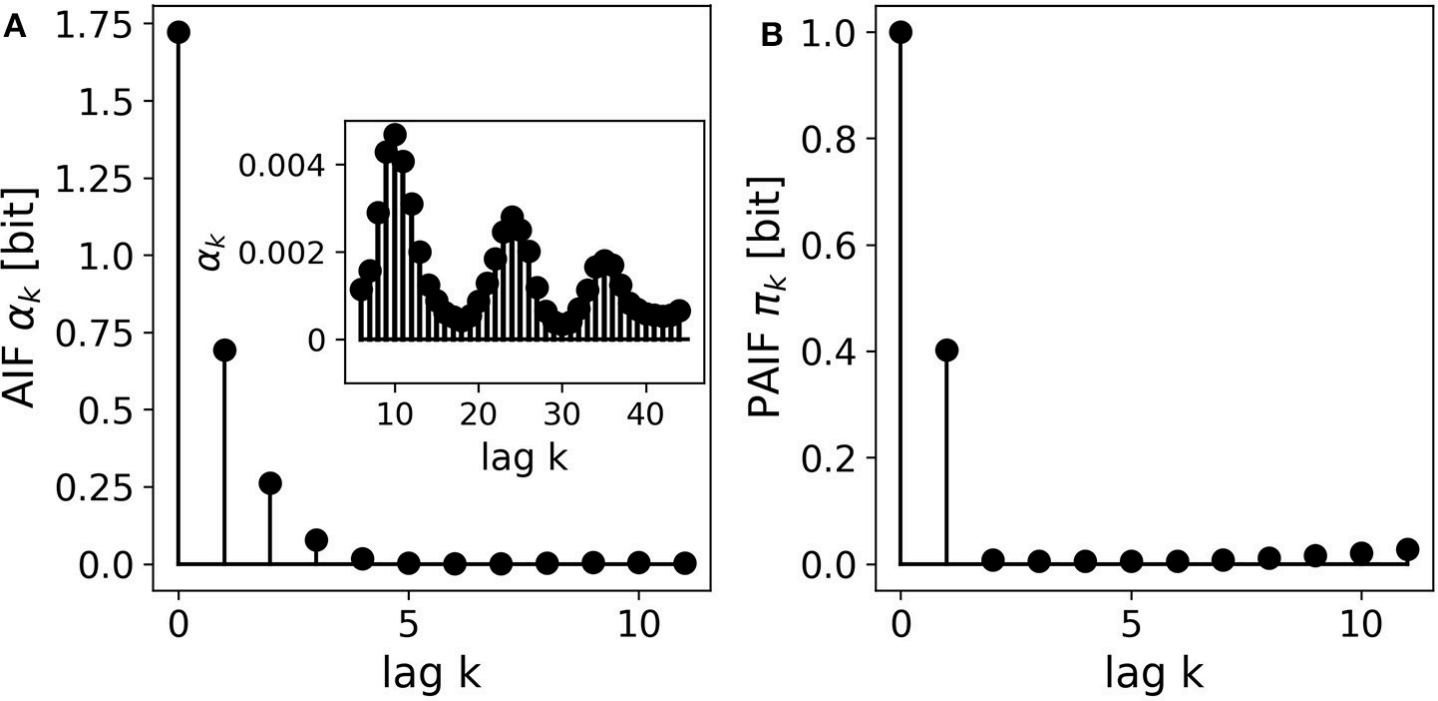
A 4-state resting state EEG microstate sequence. **(A)** The AIF shows a monotonous decay for smaller time lags *k* < 10. The inset shows the AIF for larger time lags (*k*_*max*_ = 50) and reveals periodicities that could not be predicted at shorter time scales. **(B)** The PAIF indicates a mainly first-order Markovian structure but does not allow the computation of time lags as large as the AIF due to the exponentially growing size of the associated distributions.

The EEG microstate sequence shows a more complex behavior than the other presented examples. Figure 7A shows the AIF of the 4-state sequence, which seems to decay monotonously. The inset shows further information for longer time lags up to *k* = 40. We observe several periodic peaks at these time lags, an effect that we have discussed in detail in a recent publication (von Wegner et al., 2017). The PAIF in Figure 7B shows a dominant coefficient π_1_. This finding suggests a mainly first-order Markov mechanism, and does not hint at the periodic behavior found in the AIF. Moreover, we observe increasing PAIF coefficients for larger time lags (*k* > 8). This effect is caused by finite joint entropy estimates, which suffer from an insufficient sample size, given the history length *k*, and the size of the state space, here *L* = 4.

## 4. Discussion

In the present article, an information-theoretical approach for the early diagnostic steps in symbolic time series analysis is established. In close analogy to classical time series analysis of continuous-valued random variables, a combined approach using two different measures that estimate the dependency between two time points is used. While autoinformation measures the statistical dependence between *X*_*n*_ and *X*_*n*+*k*_ directly, partial autoinformation removes the influence of the segment between both time points. The names AIF and PAIF were chosen to represent the close connection with the ACF/PACF approach. We have recently used the AIF to characterize stochastic processes and experimental EEG data (von Wegner et al., 2017, 2018), and the underlying functional can be found under the name of time-lagged mutual information in the literature. Partial autoinformation, however, is not found in the literature, to the best of the author's knowledge. Close connections to the entropy rate and the active information storage of the process, two well-studied information-theoretical quantities (Cover and Thomas, 2006; Lizier et al., 2012), are found and detailed. In particular, the newly introduced PAIF can be expressed either as the difference of two entropy rates with history lengths *k* − 1 and *k*, respectively, or as the difference of two active information storage terms with different history lengths. These relationships also assure that the PAIF coefficients approach zero in the large *k* limit.

The ability of the PAIF to identify the order of a stationary Markov process is shown analytically by re-writing the PAIF in terms of conditional entropies. A short proof shows that the PAIF coefficients of a stationary Markov process of order *M* are zero (π_*k*_ = 0) for *k* > *M*. The practical performance of the method is validated numerically, using test data with known Markov orders, and by comparison with the results of two other tests (Kullback et al., 1962; van der Heyden et al., 1998). All test examples used in this article are correctly classified by the PAIF approach, in the same way the PACF performs for continuous autoregressive processes. A close relationship between the PAIF and the conditional entropy test (van der Heyden et al., 1998) is established by re-writing both in terms of joint entropies *H*_*k*_. We found that while the conditional entropy test addresses the first-order discrete difference of *H*_*k*_ with respect to *k*, the PAIF actually tests the corresponding second-order discrete derivative. This completes the goal of establishing an information-theoretical tool analogous to classical PACF analysis.

Our experimental data examples also reveal some important limitations of the approach. The PAIF coefficients for the 4-state EEG microstate sequence (*n* = 153, 225 samples) increase for time lags above approximately *k* > 8. Comparison with Markov surrogate samples shows that this increase is due to the limited sample size, and is not a feature of the EEG data set (data not shown). The effect is easily understood by a simple numerical example. If for the same data set, we wanted to compute the PAIF coefficients for the same time lags as used in the AIF (Figure 7), joint probability distributions with *L*^*k*^ bins will occur. Thus, to extend the PAIF analysis of a *L* = 4-state process to *k* = 50, distributions with 4^50^ > 10^30^ elements have to be estimated, clearly exceeding the length of the data sample numerous times. The example also shows that this is an intrinsic limitation of the approach, as it always occurs for information-theoretical quantities involving joint entropies, and is not specific to the PAIF introduced here.

Finally, the present article exclusively deals with discrete stochastic processes. Future investigations should include the corresponding quantities for continuous random variables, and Gaussian processes in particular. For example, it has been shown in the past that for Gaussian random variables, Granger causality is equivalent to transfer entropy (Barnett et al., 2009). By analogy, it can be conjectured that the PAIF and PACF approaches are likely to be related, if not equivalent, for Gaussian processes.

It will be interesting to see further applications of the presented approach to theoretical and experimental data and to investigate further theoretical connections to other quantities already in use.

## Author contributions

FvW designed the study, performed all presented analyses, and wrote the manuscript.

### Conflict of interest statement

The author declares that the research was conducted in the absence of any commercial or financial relationships that could be construed as a potential conflict of interest.
